# Rapid Administration of High-Dose Intravenous Methylprednisolone Improves Visual Outcomes After Optic Neuritis in Patients With AQP4-IgG-Positive NMOSD

**DOI:** 10.3389/fneur.2020.00932

**Published:** 2020-09-02

**Authors:** Tetsuya Akaishi, Takayuki Takeshita, Noriko Himori, Toshiyuki Takahashi, Tatsuro Misu, Ryo Ogawa, Kimihiko Kaneko, Juichi Fujimori, Michiaki Abe, Tadashi Ishii, Kazuo Fujihara, Masashi Aoki, Toru Nakazawa, Ichiro Nakashima

**Affiliations:** ^1^Department of Neurology, Tohoku University Graduate School of Medicine, Sendai, Japan; ^2^Department of Education and Support for Regional Medicine, Tohoku University Hospital, Sendai, Japan; ^3^Department of Ophthalmology, Tohoku University Graduate School of Medicine, Sendai, Japan; ^4^Department of Neurology, National Hospital Organization Yonezawa National Hospital, Sendai, Japan; ^5^Department of Neurology, Tohoku Medical and Pharmaceutical University, Sendai, Japan; ^6^Department of Multiple Sclerosis Therapeutics, Fukushima Medical University, Fukushima, Japan

**Keywords:** neuromyelitis optica spectrum disorders, optic neuritis, steroid pulse therapy, timing, visual prognosis

## Abstract

**Objective:** The purpose of this study was to elucidate the rapid impact of high-dose intravenous methylprednisolone pulse therapy (1,000 mg/day for 3 days) on the eventual visual prognosis in patients with serum anti-aquaporin-4 immunoglobulin G (AQP4-IgG)–positive neuromyelitis optica spectrum disorders (NMOSDs) who had an attack of optic neuritis (ON).

**Methods:** Data from 32 consecutive NMOSD patients (1 male and 31 female) with at least one ON attack, involving a total of 36 ON-involved eyes, were evaluated. The following variables at ON onset were evaluated: sex, age at the first ON episode, visual acuity at nadir, visual acuity after 1 year, duration from ON onset to treatment for an acute ON attack, cycles of high-dose intravenous methylprednisolone pulse therapy for the ON attack, and cycles of plasmapheresis for the ON attack. Among the 36 ON-involved eyes, 27 eyes were studied using orbital MRI with a short-T1 inversion recovery sequence and gadolinium-enhanced fat-suppressed T1 imaging before starting treatment in the acute phase.

**Results:** In univariate analyses, a shorter duration from ON onset to the initiation of high-dose intravenous methylprednisolone pulse therapy favorably affected the eventual visual prognosis 1 year later (Spearman's rho = 0.50, *p* = 0.0018). The lesion length on orbital MRI was also correlated with the eventual visual prognosis (rho = 0.68, *p* < 0.0001). Meanwhile, the days to steroid pulse therapy and lesion length on orbital MRI did not show a significant correlation. These findings suggest that the rapidness of steroid pulse therapy administration affects the eventual visual prognosis independent of the severity of ON. In multivariate analysis, a shorter time from ON onset to the start of acute treatment (*p* = 0.0004) and a younger age at onset (*p* = 0.0071) were significantly associated with better visual outcomes.

**Conclusions:** Rapid initiation of high-dose intravenous methylprednisolone pulse therapy is essential to preserve the eventual visual acuity in patients with serum AQP4-IgG-positive NMOSD. Once clinicians suspect acute ON with serum AQP4-IgG, swift administration of steroid pulse therapy before confirming the positivity of serum AQP4-IgG would be beneficial for preserving visual function.

## Introduction

Neuromyelitis optica spectrum disorder (NMOSD) is an autoimmune-related neurological disorder that primarily causes astrocytic damage throughout the central nervous system and is characterized by the presence of serum anti-aquaporin-4 immunoglobulin G (AQP4-IgG) (1, 2). Patients with NMOSD typically present with repeated attacks of optic neuritis (ON) and/or myelitis (3, 4), and are likely to relapse without proper relapse prevention treatments, acquiring neurological disabilities accumulated in a stepwise manner (5–7). In the acute phase of attacks in NMOSD, immune suppression with high-dose intravenous methylprednisolone (IVMP) pulse therapy with or without oral tapering is the gold standard treatment at present (8–12). In addition to IVMP, plasma exchange (PLEX) and intravenous immunoglobulin (IVIg) therapy are also known to be effective for treating acute NMO exacerbations and preventing relapse (13–15). To prevent relapses in the chronic phase, mycophenolate mofetil, rituximab, azathioprine, and IVIg are being used (12, 16). Although not a standard strategy, long-term oral low-dose corticosteroids are also used as relapse prevention therapy in some facilities (17, 18). Other monoclonal antibodies, such as eculizumab, tocilizumab, satralizumab, and inebilizumab, are also known to effectively suppress autoimmunity and relapses in NMOSD, although not all these listed drugs have been approved yet (19–22).

Compared with other demyelinating neurological disorders of the central nervous system, such as multiple sclerosis (MS) or anti-myelin oligodendrocyte glycoprotein antibody (MOG-IgG)-associated demyelination, neurological disability resulting from attacks of ON in NMOSD is known to be much more severe (9, 23, 24). If untreated, it is empirically believed that up to half of the patients will eventually become wheelchair bound and/or blind (25–27). Even if properly treated with timely acute therapies and adequate relapse prevention therapies, patients with NMOSD may eventually become wheelchair bound or blind. The disease is known to have a female predominance and is associated with an increased rate of complications with other autoimmune-related diseases (i.e., Sjögren's syndrome), but there have been no established patient background factors that significantly affect the subsequent neurological disability in the disease (28, 29).

As for the manifestation of ON, patients with MOG-IgG and those with AQP4-IgG often show similar appearance on orbital MRI, often with longitudinally extensive ON lesions and swollen optic nerves in the acute phase (30), but the eventual visual prognosis with appropriate acute therapies is thought to be generally worse in AQP4-IgG-positive cases than in MOG-IgG-positive cases (31). As a result, there is an urgent need to develop an effective therapeutic strategy to preserve long-term visual outcomes in patients with AQP4-IgG-positive ON. In this study, to clarify the impact of acute therapies on the eventual neurological prognosis in NMOSD, we assessed the impacts of rapidity and total amount of acute ON treatment on eventual visual acuity (VA) in the chronic phase.

## Methods

### Study Design

**Figure 1:**
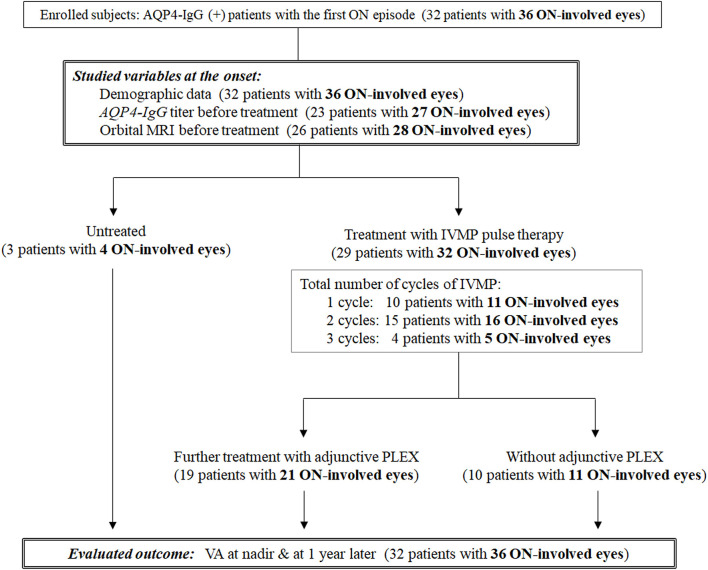
Flow diagram of the subgroup classification according to the acute therapies. A total of 32 AQP4-IgG (+) patients with a first episode of optic neuritis (ON; 36 ON-involved eyes) were enrolled. Three patients were untreated, and the remaining 29 patients (32 ON-involved eyes) were initially treated with intravenous methylprednisolone pulse therapy. A total of 19 patients (21 ON-involved eyes) were further treated with adjunctive plasma exchange. AQP4-IgG, anti-aquaporin-4 autoantibodies; IVMP, intravenous methylprednisolone pulse therapy; ON, optic neuritis; PLEX, plasma exchange; VA, visual acuity.

A total of 32 consecutive patients with AQP4-IgG-positive NMOSD with at least one ON episode who became legally blind (i.e., corrected VA ≤ 20/200, 0.1 decimal) at nadir in the acute phase were retrospectively studied. All patients were diagnosed and followed at a single university hospital in Japan between 1995 and 2019. Three other NMOSD patients whose VA at nadir was >20/200 were excluded because they had mild visual impairment; their inclusion may have biased the results. Positivity of serum AQP4-IgG and MOG-IgG was examined in all enrolled patients, and brain MRI and contrast-enhanced orbital MRI were performed to make an accurate diagnosis. There were no cases of double-positive AQP4-IgG and MOG-IgG. Only the first ON attack in each eye was evaluated; thus, relapsing ON episodes in each eye were not taken into consideration. Because four patients had ON attacks in both eyes (3 simultaneous and 1 asynchronous), a total of 36 ON-involved eyes in 32 NMOSD patients were evaluated in this study. A flow diagram of the subgroup classification according to the acute therapies is shown in Figure 1.

### Studied Variables

At each occasion of ON attack (i.e., the first ON attack in each eye), the following clinical information was comprehensively collected: sex, age, and medical history (i.e., complication of other autoimmune-related diseases), details of the acute treatment for ON, serum AQP4-IgG titer, longitudinal length of ON lesions on orbital MRI before the acute treatments, corrected VA at nadir in the acute phase, corrected VA 1 year later in the chronic phase, the required days from the ON onset to starting the acute treatments with IVMP pulse therapy, and the required days from the ON onset to starting PLEX as an adjunctive therapy.

### Contrast-Enhanced Orbital MRI

Before starting the treatment, the longitudinal length of the ON lesion on orbital MRI was evaluated in 28 of the 36 ON episodes, which was semi-quantitatively represented by the number of involved segments in the following six areas of the optic nerves: anterior orbital, posterior orbital, canalicular, intracranial, chiasmal, and optic tract (32). Involvement of the optic nerves was judged based on imaging with short-T1 inversion recovery (STIR) sequence and gadolinium-enhanced fat-suppressed T1 imaging.

### Subsequent Visual Prognosis

Corrected VA was initially measured by decimal acuities and later converted to the logarithmic minimum angle of resolution (logMAR) scale because of its statistical usability (33). The logMAR scores <0.0 were all regarded as 0.0, and logMAR scores >2.0 were all regarded as 2.0, resulting in the measured logMAR scores ranging between 0.0 (corrected decimal VA ≥ 1.0) and 2.0 (corrected decimal VA ≤ 0.01).

### Serum AQP4-IgG Titration

Before the initiation of treatment, serum AQP4-IgG titer was evaluated in 27 of the 36 ON episodes with a microscopic live cell–based assay method, using human embryonic kidney 293 (HEK293) cells expressing the human M23-AQP4 protein and Alexa Fluor 488-conjugated secondary antibody (Life Technologies, Frederick, MD, USA) (34). The titration was performed semi-quantitatively with a serial two-fold end-point dilution method (35).

### Data Analysis

Correlations between two continuous variables were evaluated with Spearman's rho, followed by a test of no correlation. When drawing the scatter plot for the days from ON onset to the initiation of acute treatment (i.e., IVMP pulse therapy, adjunctive PLEX), data from untreated patients were tentatively set to 1,000 for visual convenience after log transformation. Because the correlations were evaluated with a non-parametric statistical method, this tentative numerical conversion did not affect the results of the statistical analyses. After evaluating the correlation between each explanatory variable and the subsequent visual outcomes, multiple regression analysis was performed by employing variables of particular clinical interest and additional variables that had a significant impact (*p* < 0.10) on the subsequent visual outcomes in the univariate analysis (36). Statistical analyses were performed using IBM SPSS Statistics 22.0 (IBM, Armonk, New York, USA) and MATLAB R2015a software.

## Results

### Patient Backgrounds

Among the 32 enrolled NMOSD patients, 1 was male and 31 were female. The average ± SD of the age at ON onset (36 ON episodes) was 44.4 ± 15.8 years. The median (IQR; 25th−75th percentiles) of the serum AQP4-IgG titer was 1:8192 (1:1024–1:65,536); median (IQR) number of ON-involved optic nerve segments on orbital MRI was 3 segments (2–4 segments); median (IQR) days from ON onset to IVMP pulse therapy initiation was 9 days (4–33 days); median (IQR) number of cycles of IVMP pulse therapy in the acute phase was 2 cycles (1–2 cycles); and median (IQR) number of cycles of PLEX in the acute phase was 3 times (0–4 times). Six of the 32 patients had a clinical history of accompanying autoimmune-related diseases (rheumatoid arthritis, 2; Sjögren's syndrome, 2; systemic lupus erythematosus, 1; polymyositis, 1).

Among the 36 ON-involved eyes from 32 patients, 32 ON-involved eyes from 29 patients were treated in the acute phase with high-dose IVMP pulse therapy (1,000 mg/day) for 3 days, followed by low-dose oral prednisolone therapy for relapse prevention, whereas the remaining four ON episodes in three patients were not treated for unknown reasons. A single cycle of IVMP was administered to 11 ON-involved eyes from 10 patients, two cycles of IVMP were administered to six ON-involved eyes from 15 patients, and three cycles of IVMP were administered to five ON-involved eyes from four patients. Of the 32 ON-involved eyes from 29 patients who were treated with IVMP pulse therapy, 21 ON-involved eyes from 19 patients were later treated with adjunctive PLEX 3–6 times to achieve greater recovery in VA, and 1 of them was further treated with IVIg. Intravenous cyclophosphamide was not used in any of the enrolled subjects.

### Factors That May Affect the Visual Prognosis

Correlation coefficients between the studied candidates of prognostic variables and the subsequent visual prognosis, represented by logMAR VA a year later, are listed in Table 1. The strongest prognostic variable at ON onset was the longitudinal length of the ON lesion (rho = 0.669, *p* < 0.0001). The days from ON onset to starting IVMP pulse therapy also showed a moderate to strong positive correlation with the visual prognosis (rho = 0.502, *p* = 0.0018). Moreover, the AQP4-IgG titer (rho = 0.24, *p* = 0.23), repeated cycles of IVMP pulse therapy (rho = −0.24, *p* = 0.17), days from ON onset to starting PLEX (rho = 0.13, *p* = 0.44), or cycles of PLEX (rho = −0.16, *p* = 0.36) showed no significant correlation with the subsequent visual prognosis. The cycles of IVMP pulse therapy (rho = −0.01, *p* = 0.95), days from ON onset to starting PLEX (rho = 0.21, *p* = 0.37), or cycles of PLEX (rho = −0.25, *p* = 0.28) showed no significant correlation with the subsequent visual outcomes even when being calculated within those who received these therapies.

**Table 1:** Correlation coefficients between studied variables and visual outcome.

	**Patients (eyes)**	**Spearman's rho with VA after 1 year**	***p*-value**
Age at ON onset	*n* = 32 (36)	0.404	0.0146
AQP4-IgG titer before starting IVMP	*n* = 23 (27)	0.237	0.23
ON-involved segments in orbital MRI (0–6)	*n* = 26 (28)	0.669	<0.0001
**Treatments in the acute phase of ON**			
Days from ON onset to starting IVMP	*n* = 32 (36)	0.502	0.0018
Cycles of IVMP	*n* = 32 (36)	−0.235	0.17
Cycles of IVMP among those treated	*n* = 29 (32)	−0.013	0.95
Days from ON onset to starting PLEX	*n* = 32 (36)	0.132	0.44
Cycles of PLEX	*n* = 32 (36)	−0.156	0.36
**ON-involved eyes further treated by adjunctive PLEX after IVMP pulse therapy**			
Days from ON onset to starting PLEX	*n* = 19 (21)	0.21	0.37
Cycles of PLEX	*n* = 19 (21)	−0.25	0.28

*The p-values shown are the results of the test of no correlation*.

*AQP4-IgG, anti-aquaporin-4 autoantibody; IVMP, intravenous methylprednisolone; ON, optic neuritis; PLEX, plasma exchange; VA, visual acuity*.

**Figure 2:**
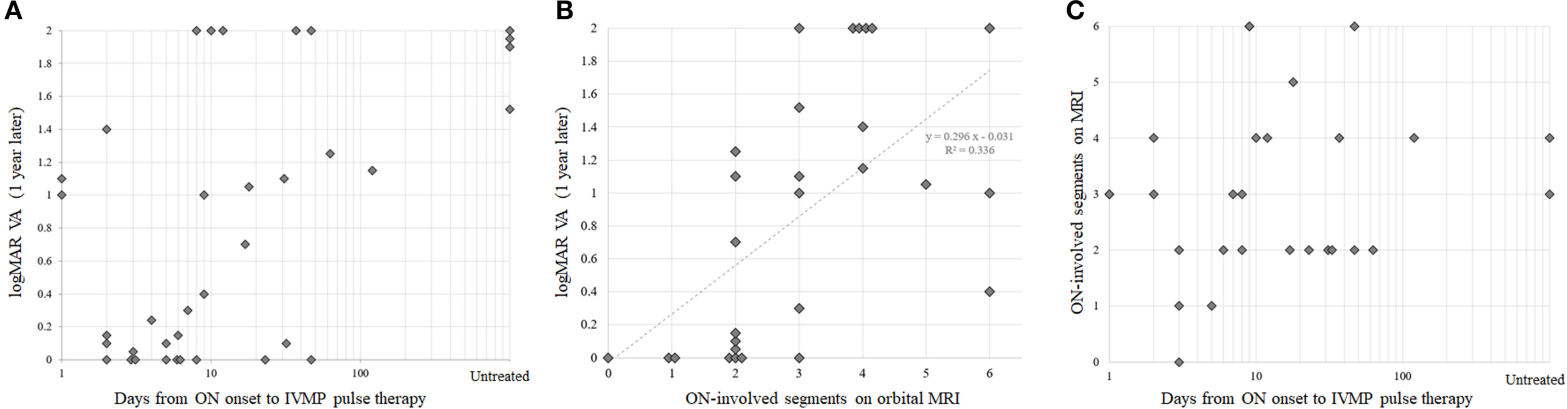
Correlations between treatment rapidity, optic neuritis (ON) severity, and visual prognosis. Scatter plots with visual prognosis and therapeutic rapidity **(A)**, with visual prognosis and ON severity **(B)**, and with ON severity and therapeutic rapidity **(C)**. Note that the horizontal axes in panels **(A,C)** are log transformed. IVMP, intravenous methylprednisolone; logMAR, logarithmic minimum angle of resolution; ON, optic neuritis; VA, visual acuity.

To visually confirm the observed correlation between rapidity of treatment in the acute phase and the subsequent visual prognosis, scatter plots with these two variables are shown in Figure 2A. Scatter plots with each of these variables and the ON-involved lesion length as a representative of ON severity are shown in Figures 2B,C. Because there was no significant correlation between rapidity of treatment and ON severity on MRI, the confounding effect of ON severity on the implied significance of rapidity of treatment in the acute phase for preserving subsequent VA was unlikely. When the partial correlation coefficient was calculated using ON-lesion length, days from ON onset to the start of IVMP, and visual outcomes at 1 year, the partial correlation coefficient between the rapidity of IVMP and visual outcomes was still statistically significant with a value of 0.437 (*p* = 0.0248, test of no correlation).

After univariate analyses, multiple regression analysis was additionally performed by employing demographic or therapeutic variables of particular clinical interest and further variables that showed *p* < 0.10 in the aforementioned univariate regression analyses. Consequently, the time from ON onset to the start of IVMP pulse therapy, cycles of IVMP pulse therapy, cycles of adjunctive PLEX, and age at ON onset were used as explanatory variables. The eventual VA after 1 year was used as the outcome variable. Shorter duration from ON onset to the start of IVMP pulse therapy [*F* = 15.81, *p* = 0.0004] and younger age at ON onset [*F* = 8.31, *p* = 0.0071] significantly contributed to better visual outcomes, whereas the number of cycles of IVMP [*F* = 2.15, *p* = 0.15] and adjunctive PLEX [*F* = 0.29, *p* = 0.60] did not. On removing the cycles of adjunctive PLEX from the explanatory variables, a shorter duration from ON onset to the start of IVMP pulse therapy [*F* = 15.98, *p* = 0.0004] and younger age at ON onset [*F* = 9.04, *p* = 0.0051] still significantly contributed to better visual outcomes, but the number of cycles of IVMP [*F* = 1.78, *p* = 0.0840] did not.

In addition to these clinical data at ON onset, the prognostic effect of other coexisting autoimmune-related diseases on eventual visual outcomes was also evaluated. The visual prognosis was suggested to be slightly worse in patients with other autoimmune-related diseases, but no statistically significant difference was observed (logMAR median 0.90 vs. 0.35; *p* = 0.32, Mann–Whitney *U*-test).

### Adjunctive Effect of PLEX

Next, to exclude the possibility of confounding effect from PLEX to the prognostic impact of early IVMP pulse therapy, we calculated the partial correlation coefficient by using days from ON onset to start IVMP, times of performed PLEX, and visual outcomes at 1 year. As a result, the partial correlation coefficient between the rapidity of IVMP and visual outcomes was also statistically significant with the value of 0.567 (*p* = 0.0004, test of no correlation), suggesting that early administration of high-dose IVMP is important to preserve the long-term visual outcomes irrespective of the addition of PLEX as an adjunctive therapy. Meanwhile, the calculated partial correlation coefficient between times of PLEX and visual prognosis was 0.184 (*p* = 0.29). Based on these results, early IVMP pulse therapy was suggested to be more important than the timing or total times of adjunctive PLEX to preserve the subsequent visual outcomes.

## Discussion

In this study, we showed that rapid initiation of treatment (i.e., IVMP pulse therapy) in the acute phase of ON in patients with AQP4-IgG-positive NMOSD significantly improved the subsequent visual outcomes 1 year later. In line with our previous findings, the visual prognosis of ON in those with NMOSD was largely affected by the ON-lesion severity, which was represented by the number of ON-involved segments on orbital MRI in the acute phase (32). Moreover, the observed impact of rapid IVMP pulse therapy on the subsequent visual outcomes was independent of the severity of the ON lesion. A similar observation was reported in several previous reports. In a report from Germany (37), an early therapeutic intervention was suggested to result in a higher complete remission rate after NMOSD attacks. Same as the present study, this previous report also demonstrated the decreased therapeutic responses in the elderly patients. Another report suggested greater preservation of retinal nerve fiber layer thickness on optic coherence tomography in patients with NMOSD who were swiftly treated with IVMP pulse therapy in the acute phase of ON (38). A more recent study (39), in which a total of 27 patients with either AQP4-IgG-positive ON (AQP4-ON) or MOG-IgG-positive ON (MOG-ON) were retrospectively enrolled for treatment-related subgroup analyses, showed that those who were treated with IVMP pulse therapy within 7 days showed better visual outcomes 3 months later than those who started treatment more than 7 days after ON onset. Together with these previous studies, the present study supports the effectiveness of timely IVMP pulse therapy in preserving long-term VA in patients with NMOSD. This finding could be hypothetically explained by irreversible severe astrocytic damage and subsequent neuronal damage, accompanied with impairments in the blood–brain barrier and complement-mediated vascular permeability, which may steadily progress without swift administration of high-dose IVMP (40). Furthermore, although the rapidity of IVMP pulse therapy was confirmed to be effective in preserving long-term VA, the total number of cycles of IVMP pulse therapy failed to show a significant effect on the subsequent visual outcomes. This is consistent with the findings of a previous study that showed no effect of repeated IVMP pulse therapy after the second cycle on subsequent neurological disability (41).

In the early 1990s, more than 10 years before the serum AQP4-IgG and MOG-IgG were measured in patients with ON, a landmark clinical trial of the Optic Neuritis Treatment Trial (ONTT) was performed (42, 43). In the ONTT, a total of 457 ON patients were enrolled and randomly allocated to each oral prednisone group (*n* = 156): IVMP + oral prednisone group (*n* = 151) and oral placebo group (*n* = 150). The result of ONTT was that patients in the IVMP + oral prednisone group showed significantly faster improvement of VA than those in the oral placebo group, but there was no significant difference in the subsequent visual outcomes between the groups both at 6 months and at 1 year. Based on these results, the general opinion among ophthalmologists and neurologists about the effectiveness of IVMP pulse therapy as an acute treatment for ON has long remained controversial. In 2004, a groundbreaking discovery came from a research group of Mayo Clinic that showed the presence of AQP4-IgG in the serum of patients who previously had “atypical” MS that exclusively presented recurrent ON and/or myelitis (1, 2). Later, reports of serum MOG-IgG came to be known to appear in many isolated ON cases (44–46). After these discoveries, serum positivity of these antibodies was retrospectively checked by using the stored serum samples from 177 of the enrolled patients in ONTT, revealing the presence of MOG-IgG only in three patients and AQP4-IgG in none of them (47). Based on this fact, ONTT can be regarded as a randomized controlled trial for the acute treatment of MS-ON and idiopathic ON (i.e., double-seronegative for MOG-IgG and AQP4-IgG). Consequently, we have to admit that the effectiveness of IVMP pulse therapy in MOG-ON and AQP4-ON to preserve the long-term (i.e., more than 6 months) visual outcomes has not been concluded to date.

**Figure 3:**
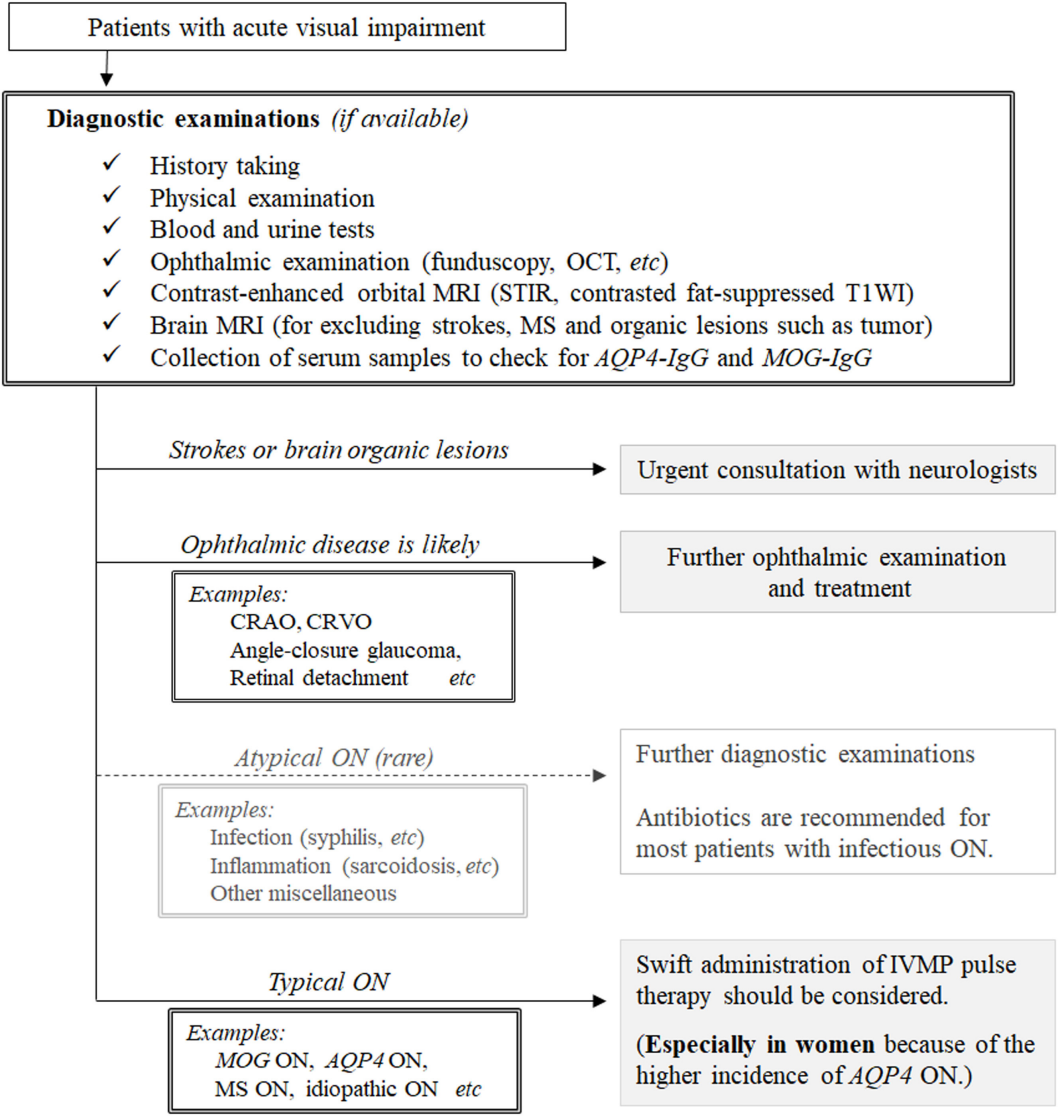
A conceivable therapeutic strategy for patients suspected of having optic neuritis. AQP4-IgG, anti-aquaporin-4 autoantibodies; CRAO, central retinal artery occlusion; CRVO, central retinal vein occlusion; DDx, differential diagnosis; HIV, human immunodeficiency virus; IVMP, intravenous methylprednisolone; MS, multiple sclerosis; MOG-IgG, anti-myelin oligodendrocyte glycoprotein antibody; ON, optic neuritis; STIR, short T1 inversion recovery; T1WI, T1-weighted imaging.

Based on the results obtained, we propose a possible therapeutic strategy for patients with undiagnosed ON, as shown in Figure 3. Before deciding to administer high-dose IVMP pulse therapy to patients suspected of ON, clinicians should exclude the possibility of infectious ON (e.g., syphilitic ON with HIV infection, Lyme ON, and tuberculous ON) because IVMP monotherapy without antibiotics may aggravate disease activity in such conditions (8, 48–50). Although the incidence of infectious ON is much lower than that of ON of other noninfectious inflammatory causes, a comprehensive examination including medical history taking, fundoscopy, imaging, and blood testing is required to correctly differentiate infectious ON before deciding on the therapeutic strategy. Once a clinician determines that the patient is unlikely to have infectious ON, the next step is to decide whether to administer high-dose IVMP pulse therapy to the patient. Currently, as discussed earlier, IVMP pulse therapy is only recommended for patients with AQP4-IgG-positive NMOSD to achieve better long-term visual outcomes; these patients are mostly female (51, 52). For patients with other diseases (i.e., MS-ON, MOG-ON, idiopathic ON), there have been no randomized controlled trials of acute treatments, and evidence regarding the use of IVMP pulse therapy to achieve better long-term visual outcomes is yet to be established. Consequently, although IVMP pulse therapy would surely quicken visual recovery from nadir VA in any of these diseases, swift administration of IVMP pulse therapy may not be mandatory to preserve the long-term visual prognosis in patients with ON without serum AQP4-IgG. However, as patients with AQP4-ON are recommended to receive IVMP pulse therapy as soon as possible after clinical onset, clinicians are often required to proceed with IVMP pulse therapy administration before obtaining the results of serological tests for serum AQP4-IgG positivity. Although the majority of patients with AQP4-ON are female, the proportions of female patients in AQP4-ON cohorts of previous studies involving different ethnicities varied to some extent between 80 and 95% (53–55). Thus, regardless of the sex of patients with ON, clinicians may consider administering IVMP pulse therapy before confirming the positivity of serum AQP4-IgG once the rare differential diagnosis of infectious ON has been ruled out or considered unlikely.

Intravenous administration is usually selected as the route of high-dose steroid (i.e., 1,000 mg/day) in the acute phase of ON, but oral administration of bioequivalent doses to high-dose IVMP may also be used as an alternative to IVMP (56). Meanwhile, low-dose oral steroid (e.g., 1 mg/kg/day) monotherapy as an acute treatment should be avoided because such an approach to the acute ON episode may not only be an insufficient immunosuppressive treatment but also increase the risk of recurrent ON in MS-ON and idiopathic ON, as suggested in ONTT (43). The serum sample for the determination of serum AQP4-IgG and MOG-IgG should ideally be collected before the administration of IVMP pulse therapy. However, serum samples should be submitted for AQP4-IgG checkup even when IVMP pulse therapy has begun before the sample collection because a recent article reported that serum AQP4-IgG titer may not significantly decrease after IVMP pulse therapy or long-term oral steroid usage, although IVMP pulse therapy may decrease the AQP4-IgG titer to some extent with a narrowly significant level (57).

As a limitation of this study, all enrolled patients with NMOSD were Asian. Further clinical studies are needed to determine whether the observed effectiveness of IVMP pulse therapy in the acute phase of AQP4-ON to improve long-term visual outcomes can be generalized to other ethnicities. In addition, because this study was performed in a retrospective manner, a randomized controlled trial with a pure cohort of AQP4-ON is desired in the future to establish high-level evidence of the impact of swiftly initiating IVMP pulse therapy in the acute phase of AQP4-ON. Furthermore, we did not consider visual field impairments in this study. As is well known, AQP4-ON patients often present with sectional visual field impairment, such as bitemporal or altitudinal hemianopia and non-central scotoma (58), and these patients may present a relatively preserved VA at the nadir (i.e., >20/200 VA) despite the disability in daily living. However, the possible bias from these cases to this study was unlikely because the excluded NMOSD patients with relatively preserved VA in the acute phase of suspected ON episodes were only 3; the visual outcomes of these patients was much better than that of others regardless of the acute treatments, and it was scientifically reasonable to exclude these patients in advance from this study that evaluated the long-term visual outcomes. Another limitation was that 10 of the 32 enrolled patients had been already evaluated for the correlation between retinal nerve fiber layer thickness and the swiftness of IVMP pulse therapy in a previous article (38). However, the achieved results in the present study was reproduced when we analyzed by using only the new 22 patients, with the calculated Spearman's rho between the swiftness of IVMP pulse therapy and the visual outcomes of 0.433 (*p* = 0.0348). Thus, apart from the previous article, the present study further reinforced the rationale of swift IVMP pulse therapy in the acute phase of AQP4-ON to preserve long-term visual outcomes. Lastly, this study failed to show the effectiveness of the rapidity and cycles of adjunctive PLEX in preserving long-term visual outcomes, but this does not contradict the effectiveness of PLEX in patients with AQP4-ON. The relatively small number of patients treated with adjunctive PLEX and possible tendency of patients who were refractory to IVMP pulse therapy to be administered adjunctive PLEX may explain why the rapidity and cycles of adjunctive PLEX failed to yield significant results in this study. Overall, a randomized trial is needed to conclude the effectiveness of adjunctive PLEX after IVMP pulse therapy in patients with AQP4-ON.

## Conclusions

In patients with AQP4-IgG-positive NMOSD, swift administration of high-dose IVMP pulse therapy is recommended to preserve the subsequent long-term visual outcomes, even before obtaining the results of tests for serum AQP4-IgG positivity. Clinicians should consider immediate administration of IVMP pulse therapy in typical ON cases without waiting to confirm AQP4-IgG positivity, especially in female ON cases because of the female predominance in AQP4-ON.

## Data Availability Statement

The raw data supporting the conclusions of this article will be made available by the authors, without undue reservation.

## Ethics Statement

The studies involving human participants were reviewed and approved by Tohoku University School of Medicine. The patients/participants provided their written informed consent to participate in this study.

## Author Contributions

TA drafted the article and created figures and tables. NH, TTake, and TN measured visual acuity at the nadir and 1 year later. TM, KF, and IN corrected MRI data. KF, MAo, TN, and IN supervised the study process. All authors contributed to the data review, interpretation of results, critical revision of article, and approval of final version of the article.

## Conflict of Interest

The authors declare that the research was conducted in the absence of any commercial or financial relationships that could be construed as a potential conflict of interest.
